# Efficient production and secretion of oxaloacetate from *Halomonas* sp. KM-1 under aerobic conditions

**DOI:** 10.1186/s13568-017-0516-9

**Published:** 2017-11-21

**Authors:** Asuka Hannya, Taku Nishimura, Isao Matsushita, Jun Tsubota, Yoshikazu Kawata

**Affiliations:** 1 Energy Technology Laboratories, OSAKA GAS Co., Ltd., 6-19-9 Torishima, Konohana-ku, Osaka, 554-0051 Japan; 2 Biomedical Research Institute, National Institute of Advanced Industrial Science and Technology (AIST), 1-8-31 Midorigaoka, Ikeda, Osaka 563-8577 Japan

**Keywords:** *Halomonas*, Oxaloacetate, Pyruvate, Poly-(*R*)-3-hydroxybutyric acid, Sodium chloride

## Abstract

The alkaliphilic, halophilic bacterium *Halomonas* sp. KM-1 can utilize glucose for the intracellular storage of the bioplastic poly-(*R*)-3-hydroxybutyric acid (PHB) and extracellular secretion of pyruvate under aerobic conditions. In this study, we investigated the effects of sodium chloride concentration on PHB accumulation and pyruvate secretion in the KM-1 strain and, unexpectedly, observed that oxaloacetate, an important intermediate chemical in the TCA cycle, glycogenesis, and aspartic acid biosynthesis, was secreted. We then further analyzed oxaloacetate productivity after changing the sodium chloride additive concentration, additive time-shift, and culture temperature. In 42-h batch-cultivation experiments, we found that wild-type *Halomonas* sp. KM-1 secreted 39.0 g/L oxaloacetate at a rate of 0.93 g/(L h). The halophilic bacteria *Halomonas* has already gained attention for industrial chemical-production processes owing to its unique properties, such as contamination-free culture conditions and a tolerance for high substrate concentrations. Moreover, no commercial scale oxaloacetate production was previously reported to result from bacterial fermentation. Oxaloacetate is an important intermediate chemical in biosynthesis and is used as a health food based on its role in energy synthesis. Thus, these data provided important insights into the production of oxaloacetate and other derivative chemicals using this strain.

## Introduction

Oxaloacetate is an important intermediate chemical in the TCA cycle, glycogenesis, and aspartic acid biosynthesis. It is used as a health food based on its role in energy synthesis; however, it can easily be decarboxylated to pyruvate in ambient water solutions (Yin et al. 2015b). Oxaloacetate was thought to have some effectiveness as a health food, such as in providing brain neuroprotection by reducing excess glutamate concentration (Zlotnik et al. 2007; Marosi et al. 2009; Boyko et al. 2012; Ruban et al. 2012) and energy production via activation of mitochondria (Haas et al. 1988). To the best of our knowledge, no commercial scale oxaloacetate production was previously reported to result from bacterial fermentation.

Recently halophilic bacteria are gaining attention for industrial chemical-production processes owing to their unique properties, such as contamination-free culture conditions and a tolerance for high substrate concentrations (Quillaguamán et al. 2010; Yin et al. 2015a). In addition, alkaliphilic bacteria are utilized to produce pure organic acids (Calabia et al. 2011; Yokaryo and Tokiwa 2014). Therefore, alkaliphilic and halophilic bacteria are appropriate candidates for industrial production of pure organic acids.

The alkaliphilic, halophilic bacterium *Halomonas* sp. KM-1 was isolated and found to store the bioplastic poly-(*R*)-3-hydroxybutyric acid (PHB) intracellularly under aerobic conditions (Kawata and Aiba 2010). In addition, the KM-1 strain secretes organic acids such as (*R*)-3-hydroxybutyric acid ([*R*]-3-HB) under microaerobic conditions (Kawata et al. 2012a) and pyruvate under aerobic conditions (Kawata et al. 2016). As mentioned previously (Kawata et al. 2012a, 2016), the KM-1 strain has potential for use in industrial fermentation applications, with some specific advantages over other microorganisms, particularly for the industrial-scale production of organic acids (Kawata et al. 2012a, 2013, 2014, 2016).

In a previous study, we found that increased buffer concentrations of sodium nitrate and sodium bicarbonate resulted in reduced PHB productivity and enhanced pyruvate secretion (Kawata et al. 2016). Passanha et al. reported that addition of NaCl affected PHB production by *Cupriavidus necator* (2014). Consistent with this, we increased the concentration of sodium chloride and found that it resulted in reduced PHB productivity and enhanced oxaloacetate secretion by the KM-1 strain. Therefore, in this study, we further explored this observation and investigated changes in oxaloacetate production resulting from different concentrations of sodium chloride, incubation times, and culture temperatures in simple batch cultivations of the KM-1 strain under aerobic conditions.

## Materials and methods

### Culture conditions

In this study, we used *Halomonas* sp. KM-1, which was previously deposited at the National Institution of Technology Evaluation as FERM BP-10995 (Kawata and Aiba 2010). KM-1 cells were routinely cultivated in modified, unsterilized SOT medium containing 12.5 g/L of sodium nitrate (Ogawa and Terui 1970; Kawata et al. 2012a) supplemented with 20% (w/v) glucose. Cells were cultured in 20 mL of medium in 200-mL Erlenmeyer flasks at 37 °C with rotational shaking at 200 rpm. In fed-batch cultivation, an additional 25 mg (0.295 mmol) of sodium nitrate was supplied at the 18, 24, 36, 42, and 48 h time points (Kawata et al. 2014, 2016).

Optimal conditions for oxaloacetate production were investigated by varying the NaCl concentration (an additional 0.3, 0.8, and 1.3 M), additive extra 0.8 M NaCl time-shift (12, 18, and 24 h), and culture temperature (30, 33, 37, and 40 °C). All experiments were performed five times.

### Analysis of PHB, glucose oxaloacetate, and pyruvate levels

To measure PHB content, samples were collected every 12 h, unless otherwise stated. PHB content was analyzed by gas chromatography (Kawata et al. 2012a; Monteil-Rivera et al. 2007) using a commercial PHB sample (Fluka, Buchs, Switzerland) as a standard. Oxaloacetate, glucose, and pyruvate content were analyzed by high-performance liquid chromatography (HPLC), as described by the manufacturer (Aminex HPX-87H; Bio-Rad, Tokyo, Japan), with commercially available, pure samples of oxaloacetate, d-glucose, and pyruvate (Wako, Osaka, Japan) used as standards.

## Results

### Effects of changing the sodium chloride concentration on the production of oxaloacetate, pyruvate, and PHB by KM-1 cells

The KM-1 strain has a relatively high tolerance for salinity until the NaCl concentration reaches 10% (w/v; 1.7 M) (Kawata et al. 2012b). In a previous study, high levels of pyruvate were observed in the secretion of KM-1 after increasing the buffer concentration of nitrate and sodium bicarbonate (Kawata et al. 2016). We suspect that increased sodium or nitrate levels induces pyruvate production, thus we simply increased the concentration of NaCl to attempt to increase pyruvate productivity; however, we unexpectedly detected oxaloacetate production (data not shown).

**Fig. 1:**
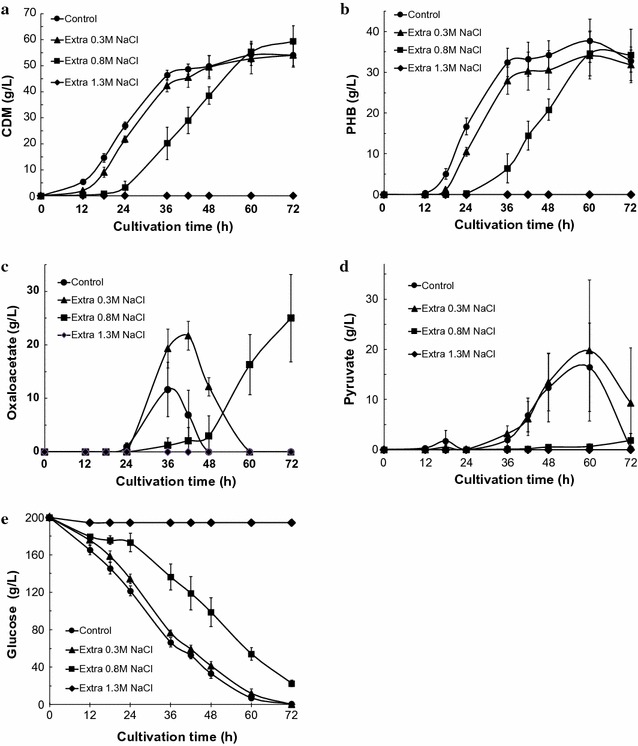
Oxaloacetate, pyruvate, and bioplastic poly-(*R*)-3-hydroxybutyric acid production by *Halomonas* sp. KM-1 with different NaCl concentrations. KM-1 cells were cultured under aerobic conditions (agitation speed: 200 rpm) at 37 °C. The medium was composed of modified SOT medium, with the control (circles), extra 0.3 M (triangles), extra 0.8 M (squares), or extra 1.3 M (diamonds) NaCl samples shown. **a** Cell dry mass (CDM), **b** intracellular bioplastic poly-(*R*)-3-hydroxybutyric acid (PHB), **c** oxaloacetate, **d** pyruvate in the medium, and **e** glucose in the medium were analyzed. The data shown represent the mean ± SD of five independent experiments

To confirm production of oxaloacetate, we examined the effects of increasing NaCl concentrations (0.3, 0.8, and 1.3 M) in simple batch cultivations of the KM-1 strain under aerobic conditions. The amounts of cell dry mass (CDM) and PHB produced by the KM-1 strain over time are shown in Fig. 1a, b. Maximal CDM production was observed after 72 h of aerobic cultivation including the addition of 0.8 M NaCl to the sample after 60 h of aerobic cultivation in the presence of 0.3 M NaCl or in the control sample, whereas minimal cell growth was observed in the sample that included an additional 1.3 M NaCl. In contrast, maximal PHB production was observed at 60 h in all samples.

In previous studies, high levels of pyruvate were obtained from samples incubated with 30.0 g/L (0.35 M) sodium nitrate in the medium; however, after the production of CDM and PHB, pyruvate stopped increasing (Kawata et al. 2016). In this case, the KM-1 strain was grown under aerobic conditions in simple batch cultivation in the presence of 12.5 g/L sodium nitrate in the initial medium and 6.25 g/L sodium nitrate in total for the fed batch. An analysis of glucose contents in the medium by HPLC revealed that glucose was consumed at a constant rate until all the glucose in the medium was consumed after 60 or 72 h of cultivation; however, the production of CDM and PHB increased until after 36 h of cultivation and mostly stopped after this period for the control and samples treated with an additional 0.3 M NaCl. In the sample exposed to an additional 0.8 M NaCl, we observed a gradual increase in CDM and PHB productivity, which reached nearly the same level as the control and the sample treated with an additional 0.3 M NaCl after 60 h (Fig. 1a, b, e). Thus, although higher pyruvate productivity was expected (Kawata et al. 2016), pyruvate production was surprisingly not observed (Fig. 1d). Unexpectedly, oxaloacetate production reached 25.0 g/L after 72 h of aerobic cultivation after the addition of an extra 0.8 M NaCl to the sample (Fig. 1c). Thus, we considered that some part of the consumed glucose was converted to oxaloacetate. We selected an additional supplement of 0.8 M NaCl for use in further experiments.

### Effects of changing the sodium chloride additive time-shift on oxaloacetate production by KM-1 cells

**Fig. 2:**
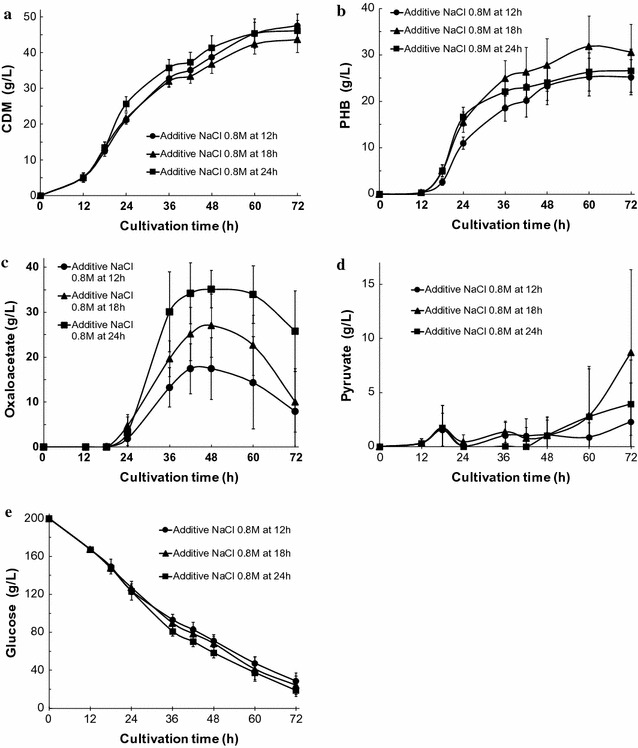
Oxaloacetate, pyruvate, and bioplastic poly-(*R*)-3-hydroxybutyric acid production by strain KM-1 using different additive NaCl time-shifts. The medium was composed of modified SOT medium. KM-1 cells were cultured under aerobic conditions (agitation speed: 200 rpm) at 37 °C, with addition of 0.8 M NaCl at 12 h (circles), 18 h (triangles), or 24 h (squares). **a** Cell dry mass (CDM), **b** intracellular bioplastic poly-(*R*)-3-hydroxybutyric acid (PHB), **c** oxaloacetate, **d** pyruvate in the medium, and **e** glucose in the medium were analyzed. The data shown represent the mean ± SD of five independent experiments

Next, we examined whether changes to the NaCl additive time-shift affected oxaloacetate productivity in simple batch cultivations of KM-1 cells under aerobic conditions. Changes in CDM, PHB, oxaloacetate, and pyruvate production as well as glucose consumption observed over time in KM-1 cells are shown in Fig. 2a–e. For samples, additions of 0.8 M NaCl were made at 12, 18, and 24 h; the amounts of CDM and the amount of glucose consumption were similar until the 72-h culture time point. Until after 60 h of cultivation, pyruvate production was minimal (less than 2.9 g/L) for all samples; even at the 72-h time point, they were controlled to less than 8.7 g/L. Thus, pyruvate production was substantially repressed to low levels. Maximal oxaloacetate production was observed by all samples after 48 h of cultivation and was found to decrease gradually until 72 h of cultivation, especially for a sample in which additional NaCl was added at 24 h after cultivation and showed the highest oxaloacetate production at 35.1 g/L in the medium (Fig. 2c). Thus, we used this condition of sodium chloride addition at the 24-h time point for use in further experiments.

### Effects of changing the culture temperature on oxaloacetate production by KM-1 cells

**Fig. 3:**
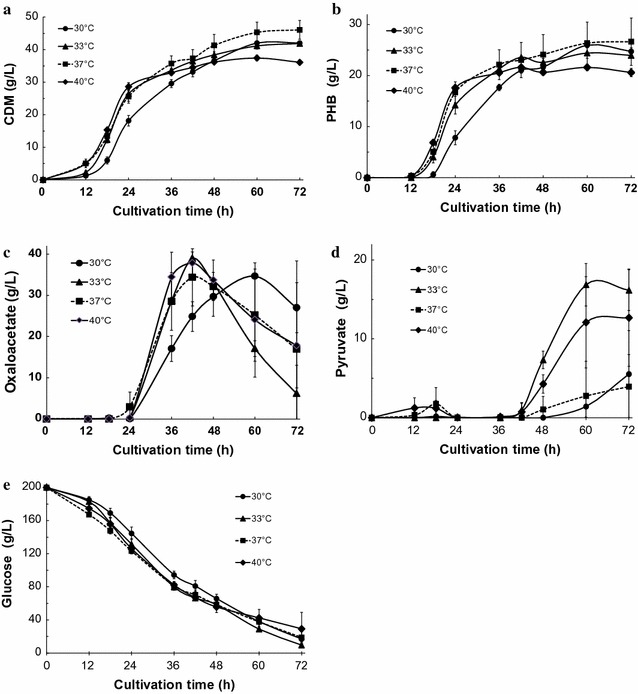
Oxaloacetate, pyruvate, and bioplastic poly-(*R*)-3-hydroxybutyric acid production by *Halomonas* sp. KM-1 under varying culture temperatures. The medium was composed of modified SOT medium. KM-1 cells were cultured under aerobic conditions (agitation speed: 200 rpm) at 30 °C (circles), 33 °C (triangles), 37 °C (squares; dotted line in Fig. 2), or 40 °C (diamonds), with addition of extra 0.8 M NaCl at 24 h. **a** Cell dry mass (CDM), **b** intracellular bioplastic poly-(*R*)-3-hydroxybutyric acid (PHB), **c** oxaloacetate, **d** pyruvate in the medium, and **e** glucose in the medium were analyzed. The data shown represent the mean ± SD of five independent experiments

Finally, we examined whether changes in the culture temperature affected oxaloacetate productivity in simple batch cultivations of KM-1 cells under aerobic conditions (Fig. 3a–e). For samples cultured at 30, 33, 37, and 40 °C, the amounts of CDM, PHB, and glucose consumption were a little less in the sample cultured at 30 °C than that in samples cultured at 33, 37, or 40 °C until the 36-h cultivation time point (Fig. 3a, b, e). Moreover, CDM and PHB levels were repressed compared to that in the control sample in which NaCl was not added, especially for samples after 36 h of cultivation (Figs. 1a, b, 3a, b, e). Instead of increasing the amount of CDM and PHB, high levels of oxaloacetate were observed, such as after 42 h of cultivation, which yielded 39.0 g/L at 33 °C, 37.9 g/L at 40 °C, 34.4 g/L at 37 °C; after 60 h of cultivation, 34.7 g/L was produced at 30 °C, respectively (Fig. 3c). The pyruvate levels also increased after 48 h. At the same culture time point at which maximal oxaloacetate production was obtained, pyruvate production was found to be negligible—less than 1.4 g/L until after 42 h of cultivation (Fig. 3d). Schügerl (2000) suggested that organic acid accumulation may suppress growth and byproduct formation during organic acid fermentation. The alkaliphilic, halophilic bacterium *Halomonas* sp. KM-1 have already shown to store PHB intracellularly (Kawata and Aiba 2010), and secretes organic acids such as (*R*)-3-HB under microaerobic conditions (Kawata et al. 2012a), and pyruvate under aerobic conditions (Kawata et al. 2016) with commercial productivity. Thus, we report a novel method of oxaloacetate production that should produce sufficient quantities for commercial application.

## Discussion

In this study, changes in oxaloacetate secretion by strain *Halomonas* sp. KM-1 were investigated in the presence of different NaCl concentrations, additive NaCl time-shifts, and culture temperatures in simple batch cultivations under aerobic conditions. In the case of *Cupriavidus necator*, a concentration of 0.15 M NaCl promoted PHB production, whereas a slightly higher concentration of 0.26 M NaCl repressed PHB production (Passanha et al. 2014). In a previous study, a high level of pyruvate secretion and suppression of PHB production by KM-1 was observed with increased sodium nitrate and sodium bicarbonate buffer concentrations (Kawata et al. 2016). To clarify the relationship among pyruvate production, PHB suppression, and sodium nitrate concentration, we increased the concentrations of sodium chloride, finding that pyruvate productivity was not increased, whereas oxaloacetate secretion by KM-1 cells was unexpectedly observed (Fig. 1c, d). Based on this phenomenon, the sodium chloride concentration is associated with the production of oxaloacetate and pyruvate as well as PHB suppression. Moreover, the association between oxaloacetate, PHB, and pyruvate production and sodium chloride concentration observed in this study has not been previously described.

The KM-1 strain is routinely cultured at pH 9.4, with a sodium carbonate buffer concentration of 0.2 M and a sodium chloride concentration of 1.0 g/L (17.1 mM) (Kawata and Aiba 2010; Kawata et al. 2014). However, the KM-1 strain has a relatively high tolerance for salinity until the NaCl concentration reaches 10% (w/v; 1.7 M) (Kawata et al. 2012b).

Some of the methods previously studied for the production of oxaloacetate are as follows. Krebs and Eggleston reported oxaloacetate synthesis from pyruvate and carbon dioxide using minced pigeon liver (1940). Enzymatic conversion of the tartaric acids from oxaloacetate (Shilo 1957) and production of oxaloacetate and other organic acids from glucose using cancer tissue culture (Abdel-Tawab et al. 1959) were also reported. Recently, improvements in oxaloacetate production have been attempted by engineering *Escherichia coli* to overexpress codon-optimized PEPC genes from *Dunaliella salina* (Park et al. 2013) and *Photobacterium profundum* SS9 (Park et al. 2014). Production of oxaloacetate and its derivative acids were also reported using a gram-positive bacterium (Murase et al. 2006). In our study, oxaloacetate production by the KM-1 strain, both in terms of the quantity and rate, was relatively high compared to other methods used for the industrial production of oxaloacetate.

Our method for oxaloacetate production may have advantages, particularly for industrial-scale oxaloacetate production. As an alkaliphilic and halophilic bacterium, *Halomonas* sp. KM-1 was grown under moderately alkaline conditions (pH 9.4) and in the presence of moderate to high salinity (0.2–1.0 M). Moreover, the KM-1 strain does not require sterilization of culture-modified SOT medium, an inexpensive, simple, and chemically defined medium. In addition, the KM-1 strain is neither pathogenic nor recombinant; thus, no strict safety regulations are in place concerning the culture of this strain, and the KM-1 strain can be cultured at simple facilities with a low running cost. In addition, during the industrial production of oxaloacetate, the presence of organic acids in the medium may interfere with oxaloacetate purification. With the exception of pyruvate, we rarely observed peaks by HPLC analysis that were indicative of the presence of other organic acids from the KM-1 strain (data not shown). Thus, the KM-1 strain may have economic advantages for industrial oxaloacetate production.

**Fig. 4:**
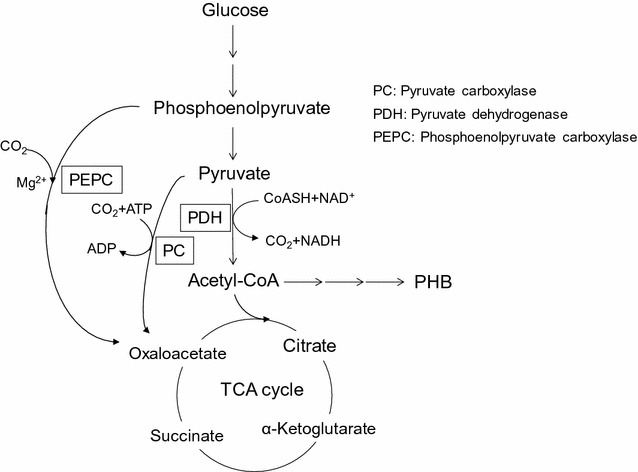
Metabolic pathway of butanoate metabolism in *Halomonas* sp. KM-1

In this study, after the addition of NaCl, the speed of CDM and PHB production decreased; in contrast, the glucose consumption rate was not changed (Fig. 3a, b, e). If the synthesis of PHB is stopped, concurrent production of pyruvate and acetate, which would be synthesized from acetyl-CoA, was also predicted to occur. However, in our study, pyruvate and acetate secretion were minimally observed (Fig. 3d, data not shown). Instead, a rapid increase in oxaloacetate production was observed (Fig. 3c). In a former study, pyruvate secretion was observed when sodium nitrate concentration was increased; moreover, CDM and PHB production was found to simultaneously stop (Kawata et al. 2016), suggesting that neither oxaloacetate producing anaplerotic pathways (PEP to oxaloacetate by PEPC or pyruvate to oxaloacetate by PC) (Fig. 4) (Vuoristo et al. 2016) were activated. In this study, the effect of sodium chloride addition seemed to activate anaplerotic pathways to secret excess amounts of oxaloacetate (Fig. 3c), but the mechanism underlying activation of oxaloacetate production, especially the anaplerotic pathway for oxaloacetate production, is unclear. In the case of amino acid production, *Corynebacterium glutamicum* also produces oxaloacetate from pyruvate and carbon dioxide using the anaplerotic pathway (Delaunay et al. 1999; Peters-Wendisch et al. 2001; Shirai et al. 2007; Sato et al. 2008), especially under biotin limiting conditions; moreover, overexpression of PEPC is crucial for glutamate production via oxaloacetate (Sato et al. 2008). Prior to 48 h of cultivation, oxaloacetate secretion was observed at high concentrations as opposed to pyruvate secretion, which was scarcely observed. After 48 h of cultivation, the glucose consumption was still as active as before, whereas oxaloacetate synthesis decreased and pyruvate secretion started to be detectable (Fig. 3c–e). Thus, these findings suggest that oxaloacetate secretion through anaplerotic pathways (PEP to oxaloacetate by PEPC) is more likely to be the pathway involved. In addition, the KM-1 strain was induced to secrete oxaloacetate by application of excess sodium chloride. Since this type of efficient oxaloacetate production system has not been well studied, additional studies are needed to clarify the mechanisms mediating oxaloacetate secretion under these conditions.

The KM-1 strain has a relatively high tolerance for salinity until the NaCl concentration reaches 10% (w/v; 1.7 M) (Kawata et al. 2012b). Thus, the wild-type KM-1 strain exhibits a natural tolerance of moderately high osmolality; accordingly, a substrate concentration of up to 20% glucose can be used to facilitate high oxaloacetate production. In this study, the alkaliphilic, halophilic bacterium *Halomonas* sp. KM-1 secreted 39.0 g/L oxaloacetate at a rate of 0.93 g/(L h) after 42 h of aerobic batch cultivation in sodium chloride- and glucose-rich medium, without sterilization. Oxaloacetate production was closely related to the sodium chloride concentration. Notably, this mechanism has not been published previously. Oxaloacetate production in this study occurred at a relatively high level, and the KM-1 strain provided several advantages, such as a contamination-free culture and the capacity for high substrate concentrations. These results suggest that the KM-1 strain may be a suitable candidate for future industrial oxaloacetate production.

## Abbreviations

PHB: poly-(*R*)-3-hydroxybutyric acid
(*R*)-3-HB: (*R*)-3-hydroxybutyric acid
SOT: Spirulina–Ogawa–Terui
PEP: phosphoenolpyruvate
PDC: pyruvate dehydrogenase complex
HPLC: high-performance liquid chromatography
PEPC: phosphoenolpyruvate carboxylase
PC: pyruvate carboxylase
